# Resting after learning or repeating the learned?

**DOI:** 10.3758/s13423-025-02820-4

**Published:** 2025-12-15

**Authors:** Markus Martini, Tom Mercer, Luis Gutmann, Stephan Frederic Dahm, Robert Marhenke, Pierre Sachse

**Affiliations:** 1 Department of Psychology, University of Innsbruck, Universitätsstraße 5–7, 6020 Innsbruck, Austria; 2 School of Education and Psychology, University of Wolverhampton, Wulfruna Street, Wolverhampton, West Midlands WV1 1LY UK; 3 Department of Information Systems, University of Innsbruck, Universitätsstraße 15, 6020 Innsbruck, Austria; 4 School of Psychology, Centre for Innovation in Mental Health, University of Southampton, B44 University Rd, Southampton, SO17 1PS UK

**Keywords:** Wakeful resting, Repetition, Memory consolidation, Social media

## Abstract

The formation of new memories can be influenced by the activities that immediately follow learning. Previous research has shown that several minutes of wakeful resting after learning can enhance memory retention compared to engaging in unrelated tasks, likely by facilitating memory consolidation. However, the relative efficacy of wakeful resting compared to other potentially memory-enhancing post-learning activities, like post-learning repetition, remains unclear. We conducted two experiments examining the effects of wakeful resting and repetition on memory retention. Participants studied three auditorily presented word lists. Each word list was immediately followed by one of three 7-min post-learning interventions: (1) repetitive listening to the word list, (2) wakeful resting, or (3) engaging in a new task. Across both experiments, memory performance was highest in the repetition condition, followed by the wakeful resting condition, with the lowest performance observed in the distraction condition. These findings were observed 12 min after acquisition and remained stable after 1 day. Our results highlight the differential effects of post-learning activities on memory retention. Wakeful resting is an effective and easy-to-implement strategy for facilitating memory consolidation, but in direct comparison, repetition appears to be even more effective for optimising memory retention.

**Supplementary Information:**

The online version contains supplementary material available at 10.3758/s13423-025-02820-4.

## Introduction

A brief period of wakeful resting immediately following new learning can enhance memory retention (Dewar et al., 2007; Mednick et al., 2011; Tambini & Davachi, 2019; Wamsley, 2019). Thus, wakeful resting is an effective and easily implementable strategy to enhance memory. While many studies investigated the efficacy of wakeful resting in supporting memory retention compared to various interfering activities, no study to date has directly compared its efficiency with other post-learning activities known to enhance memory retention (Wamsley, 2022). Consequently, it remains unclear whether wakeful resting is the optimal strategy to promote memory retention or if the post-learning period could be more effectively utilized for alternative activities, such as simply repeating the previously learned material.

### *Wakeful resting*

Behavioral studies have demonstrated that a period of wakeful resting immediately after learning – where participants rest with their eyes closed – enhances memory retention, while engaging in a distracting task after learning has comparably disruptive effects (Craig et al., 2015; Dewar et al., 2007, 2012; Dewar et al., 2012; Humiston & Wamsley, 2018; Martini et al., 2020a, b; Mercer, 2015; for conflicting findings, see Martini et al., 2020b; Varma et al., 2017). For instance, a 10-min wakeful resting period after learning a word list led to superior recall performance compared to post-learning activities such as watching TV, listening to the radio, or searching for errors in pictures (Dewar et al., 2007). During wakeful resting, the attentional focus is primarily internally oriented, allowing the mind to wander while task-oriented thoughts are reduced (Brokaw et al., 2016). This offline state, characterized by reduced external interference and limited formation of new memories, is thought to provide conditions similar to sleep, allowing certain memory consolidation processes to occur (Dudai et al., 2015; Mednick et al., 2011; Wamsley, 2022). Memory consolidation processes like repeated memory reactivation during post-encoding rest periods are thought to strengthen new memory traces, increasing the likelihood of their successful retrieval (Carr et al., 2011; Schuck & Niv, 2019; Tambini & Davachi, 2019; Wamsley, 2019). In contrast, activities that involve encoding new memories immediately after learning may disrupt reactivation processes, thereby reducing recall performance for previously learned information (Dudai et al., 2015; Mednick et al., 2011; Wixted, 2004). The beneficial effects of wakeful resting compared to task-focussed activities have been observed across different age groups (children: Fatania & Mercer, 2017; Martini et al., 2019; younger and older adults: Craig et al., 2016; Dewar et al., 2012), learning contents (verbal: Dewar et al., 2012; visuo-spatial: Craig et al., 2015), post-learning distractor tasks (Dewar et al., 2007), and retention intervals (minutes: Mercer, 2015; days: Dewar et al., 2012). A meta-analysis by Humiston et al. (2019) further supports this, reporting a moderate effect size (*d* =.38) for the benefits of wakeful resting on verbal memory. While this demonstrates that wakeful resting can enhance memory performance, other evidence suggests that this effect is unlikely to result from explicit rehearsal of the previously learned information during the rest period (see Tambini & Davachi, 2019). It is assumed that stimulus-related thoughts during rest periods are more spontaneous in nature rather than intentional, indicating that memory consolidation and reactivation processes during resting periods are not driven by explicit rehearsal (Wamsley, 2019).

## The present study

Findings from wakeful resting studies suggest that new memories benefit when learning is followed by several minutes of wakeful resting, compared to when attention is immediately directed to a new task. However, while the efficacy of brief periods of wakeful resting in supporting memory retention is well established, no research to date has explicitly investigated its effectiveness directly compared to other memory-promoting post-learning activities. One of the most obvious learning strategies is repetition of the previously learned material. The positive effects of repetition on memory are well established in the scientific literature, demonstrating that repeated exposure to a to-be-remembered stimulus enhances memory performance (Crowder, 1976; Deese, 1960; Gregg, 1976; Hebb, 1961; Kahana et al., 2024; Popov & Reder, 2020; Ward et al., 2003). These benefits have been observed across a variety of memory tasks, including free recall, recognition, and cued recall (e.g., Challis & Sidhu, 1993; Reder et al., 2016). A widely accepted view is that through repetition the information that was formed during the first encoding is strengthened from a short-term memory trace into a more stable long-term memory trace – a process described by cumulative-strength models (e.g., Murdock, 1982; Reder et al., 2000; Wickelgren, 1972; but see also Kuhl & Anderson, 2011; Musfeld et al., 2023). Recall performance is thereby a function of strength, and increasing the strength through repetition increases memory performance.

In this study, we sought to determine whether a brief period of wakeful resting immediately after learning would have similar effects on memory retention compared to a period of immediate repetition of the learned material. Specifically, we asked: Is it more effective to study and then rest for a few minutes, or to study and subsequently spend those minutes repeating the learning material? In our study, participants were asked to learn three word lists. Immediately after learning each word list, they underwent one of three post-learning conditions: (1) repetitive listening to the word list (repetition condition), (2) wakeful resting (wakeful resting condition), or (3) engaging in a new task (distractor condition). Memories for the word lists were tested immediately following the post-learning conditions and again after 1 day. We hypothesized that repeated presentation of the word list should result in the highest memory performance, and wakeful resting should lead to better memory performance than engaging in a new task. Alternatively, we considered the possibility that wakeful resting might confer memory benefits comparable to those observed with repeated presentation, suggesting that the two post-learning conditions could be similarly effective for supporting memory retention.

## Experiment 1

### Method

#### Participants

Forty-five participants took part in the experiment in exchange for course credit. Seven participants only completed Session 1. Missing recall data from these seven participants for Session 2 were imputed using the predictive mean-matching procedure in the R mice package, employing a single imputation approach (van Buuren & Groothuis-Oudshoorn, 2011). Two participants were excluded from analyses due to failing to achieve a minimum memory performance of 10% on the first recall. The analyzed data set consisted of 43 participants (27 female, 16 male; mean age = 21.8 years, *SD* = 2.4 years, age range = 19–29 years). All participants gave informed consent to take part in the study. A power analysis (Faul et al., 2007) indicated that a sample size of 44 participants would be sufficient to detect a medium effect of *f* = 0.25, with a power of 95%, a correlation of *r* =.6 among repeated measures, and a significance level of 0.05.

#### Materials and procedure

**Fig. 1:**
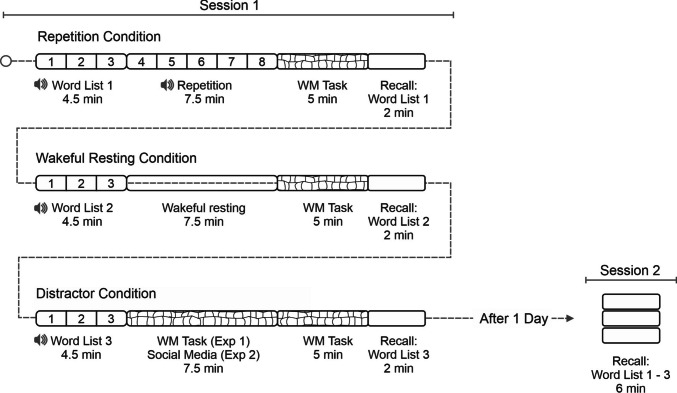
Experimental procedure. The experiment consisted of two sessions, separated by 1 day. Session 1 included three word-list-learning phases. The critical manipulation occurred during a 7.5-min phase immediately following word-list learning. During this post-learning phase, participants either continued listening to the word list (repetition condition), wakefully rested (wakeful resting condition), performed a working memory task (Experiment 1), or used social media (Experiment 2) as distractor conditions. This was followed by a 5-min working memory (WM) task and a free-recall test of the word list. In Session 2, after 1 day, participants recalled all three word lists again. The order of the post-learning conditions was counterbalanced across participants

The experiment included two testing sessions, separated by 1 day (Fig. 1). We applied a repeated-measures design, with the within-subject factors Post-Learning Condition (repetition vs. wakeful resting vs. working memory) and Recall Time (12 min vs. 1 day). In Session 1, a maximum of four participants were tested per session. Each participant's workspace was separated by partitions on the front, left, and right to ensure privacy. Participants were seated at least 2 m apart and lighting conditions were kept constant. The experiment was programmed using PsychoPy (version 2021.2.3; Peirce et al., 2019), with Session 2 conducted online on Pavlovia.org.

#### Session 1

As illustrated in Fig. 1, Session 1 consisted of three consecutive word-learning phases. Each word-learning phase consisted of (a) an auditory presentation of one of three word lists, repeated three times; (b) a 7.5-min post-learning condition, during which participants either (i) listened to the word list repeated five additional times, (ii) wakefully rested, or (iii) performed a working memory task; (c) a 5-min working memory task; (d) a free recall of the word list.

##### Word lists

The three word lists consisted of 30 German words each (90 words in total), taken from the Berlin Affective Word List Reloaded (BAWL-R; Võ et al., 2009). The words were selected on the basis of the following dimensional ranges: valence: −1.1 to 1.1; imaginability: 3.5 to 5.25; arousal: 1.5 to 3.25. The words were bisyllabic, consisting of five to seven letters (e.g., “Person [person]”, “Truhe [chest]”, “Labor [laboratory]”, “Stempel [stamp]”, “Lehrer [teacher]”). The words were presented in a pseudo-randomized order to avoid semantic or phonetic clustering. Words were presented acoustically by a female voice through headphones, with each word presented for approximately 1 s, followed by a 2-s inter-stimulus interval. The computer screen remained black during word presentation.

A preliminary study was conducted with 16 participants to ensure similar retention rates across the three word lists. This test was conducted online, with each participant randomly assigned to listen to one of the three word lists three times. Participants were then asked to immediately recall the words. There were no significant differences in the average number of words recalled across the word lists, *F*(2, 13) =.06, *p* = 0.945 (word list 1: *n* = 6, *M* = 17.5, *SD* = 6.1; word list 2: *n* = 5, *M* = 17, *SD* = 6; word list 3: *n* = 5, *M* = 18.2, *SD* = 4.6).

##### Post-learning conditions

In the *repetition condition*, participants listened to the word list eight times without interruption, instructed to memorize the words. In the *wakeful resting condition,* participants listened to the word list three times without interruption and were instructed to memorize the words. Immediately after word learning, participants were asked to rest quietly in a relaxed sitting position with their eyes closed, with the experimenter resting alongside them. During the wakeful resting phase, participants wore no headphones, and the computer screen was black. In the *working memory condition*, participants listened to the word list three times without interruption and were instructed to memorize the words. Word learning was immediately followed by performing an operation span task (Stone & Towse, 2015). In this task, participants were presented with an alternating sequence of to-be remembered numbers (ranging from 1 to 99) and arithmetic equations (e.g., 5 + 2 = 7). Participants had to encode the numbers for later serial recall and to judge the correctness of each equation. Each trial began with a visual start signal presented for 2.5 s. Immediately after the start signal, a number was displayed for 1.25 s, followed by an equation. The equation was replaced by a blank screen after participants responded (left arrow key = incorrect, right arrow key = correct), with no time limit for the responses. There was a pool of 3,000 equations, including an equal number of additions, subtractions, multiplications, and divisions. Half of the equations were correct (e.g., 10/5 = 2) and half were incorrect (e.g., 3*40 = 30), randomly selected on each trial from the total number of equations. The number of alternating sequences of numbers and equations continuously increased from 3 to a maximum of 8. Participants received brief feedback immediately after each response to an equation and each number typed in the recall phase, with a green check mark for a correct response, and a red cross for an incorrect response, each lasting 500 ms.

##### Five-minute working memory task

All post-learning conditions were followed by a 5-min working memory task, using the same operation span task as in the distractor condition (Stone & Towse, 2015). This task ensured that recall of the word list was preceded by the same task, irrespective of the post-learning condition.

##### Recall

Immediately following the 5-min working memory task, participants were asked to write down as many words as they could recall from the previously presented word list, in any order, on a blank sheet of paper within 2 min.

##### Subjective ratings

At the end of each post-learning condition, participants were asked to rate how much they thought about the words during the respective post-learning condition using a 5-point Likert scale, ranging from 1 = “not at all” to 5 = “the whole time”.

#### Session 2

A second memory test conducted online after 1 day, between 19 and 31 h after the first memory test (*M* = 25 h). Participants were instructed to type as many words as they could recall from all of the three word lists learned the previous day, in any order, within 6 min using an open text entry field.

### Results

Data were analysed using JASP (JASP Team, 2024*, Version 0.19*).

#### Memory performance

**Fig. 2:**
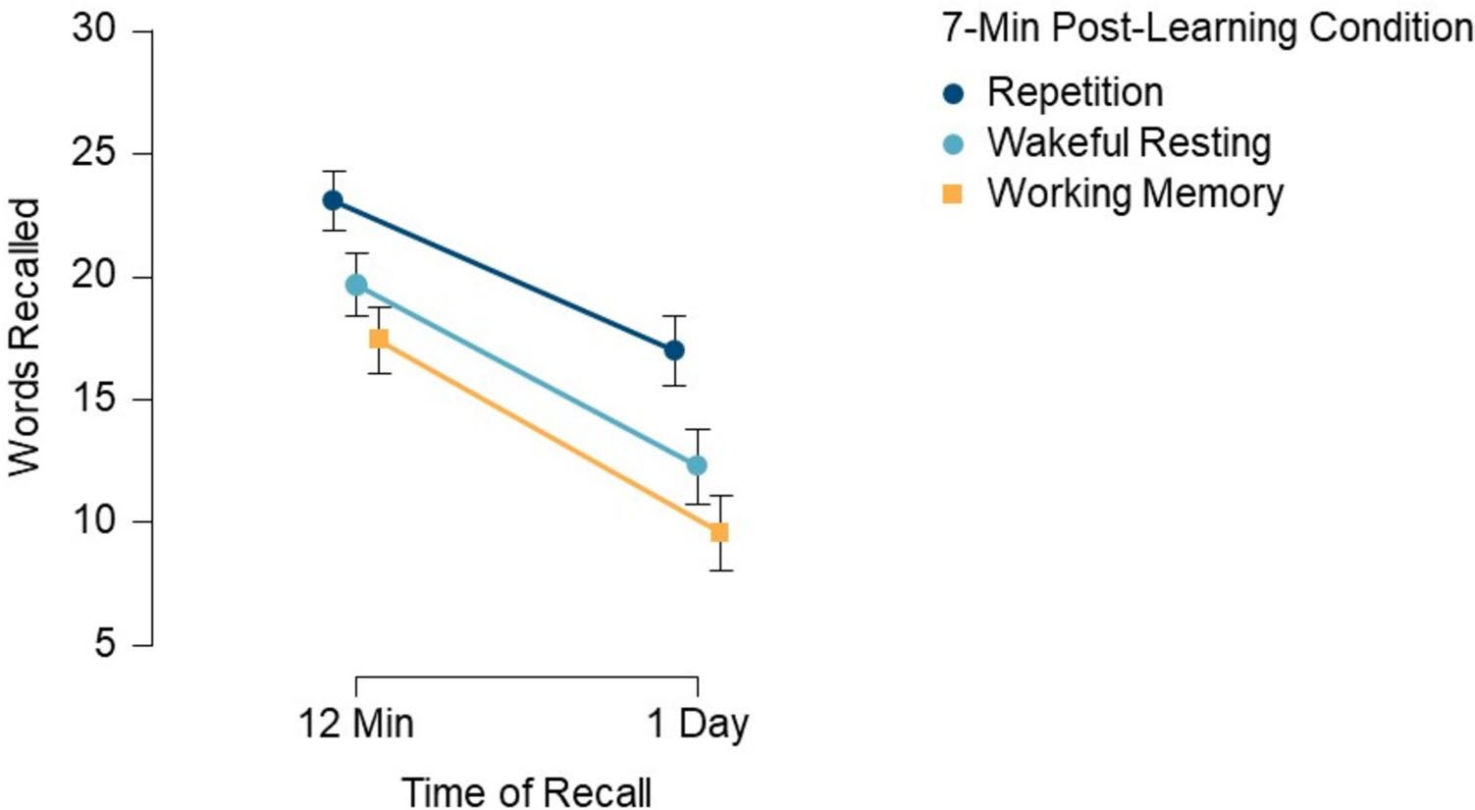
Results from Experiment 1. Mean number of correctly recalled words (maximum = 30 words) as a function of post-learning condition (repetition vs. wakeful resting vs. working memory) and time of recall (12 min vs. 1 day). Error bars represent 95% confidence intervals

Figure 2 shows the mean memory performance at the two recall times across the three post-learning conditions, presenting the raw number of correctly recalled words.

A 3 × 2 repeated-measures ANOVA was conducted with the within-subject factors of Post-Learning Condition (repetition, wakeful resting, working memory) and Time of Recall (12 min, 1 day). Participants' memories for the words differed significantly between the three post-learning conditions, *F*(2, 84) = 27.77, *p* <.001, *η*_p_ =.40. Contrast analyses revealed that participants retained significantly more words in the repetition condition compared to the wakeful resting condition, *t*(42) = 4.97, *p* <.001, *d* = 0.60, and significantly more words in the wakeful resting condition compared to the working memory condition, *t*(42) = 2.7, *p* =.010, *d* =.36 (Fig. 2). Retention dropped significantly in all three post-learning conditions over the 1-day delay, *F*(1, 42) = 166.55, *p* <.001, *η*_p_ =.80. Memory performance in the three post-learning conditions did not interact with the time of recall, indicating that memory performance differences between the three post-learning conditions were sustained across the 1-day delay, *F*(2, 84) = 2.35, *p* =.101, *η*_p_ =.05. ANOVA results without imputed data can be found in Table S1 (Online Supplementary Material).

#### Subjective ratings

Participants’ subjective ratings of how often they thought about the words differed significantly between the three post-learning conditions, *Χ*^2^(2) = 9.05, *p* =.011, Kendall’s W =.11. Conover’s post hoc comparisons revealed that ratings did not significantly differ between the repetition condition (*M* = 3.42, *SD* = 1.33) and the wakeful resting condition (*M* = 3.35, *SD* = 1.34), *T*(84) =.59, *p*_holm_.554. Ratings were significantly higher in the repetition condition compared to the working memory condition (*M* = 2.95, *SD* = 1.46), *T*(84) = 2.97, *p*_holm_ =.012, and higher in the wakeful resting condition compared to the working memory condition, *T*(84) = 2.38, *p*_holm_ =.040.

Correlations between participants’ subjective ratings and recall performance after 12 min in the respective post-learning condition were small to medium (Cohen, 1992; repetition condition: *r*_Spearman_ =.22, *p* =.163; wakeful resting condition: *r*_Spearman_ =.33, *p* =.03; working memory condition: *r*_*Spearman*_ =.4, *p* =.008). Correlations between participants’ subjective ratings and recall performance after 1 day in the respective post-learning condition were medium (repetition condition: *r*_Spearman_ =.29, *p* =.06; wakeful resting condition: *r*_Spearman_ =.25, *p* =.113; working memory condition: *r*_Spearman_ =.35, *p* =.02).

## Experiment 2

Experiment 2 was identical to Experiment 1, except that the participants used social media instead of performing a working memory task after learning in the distraction condition. The aim of replacing the working memory task with social media usage was to replicate the findings of Experiment 1 in a more ecologically valid (distractor) context, one that better reflects everyday post-learning activities. Social media is a pervasive aspect of daily life, particularly among younger adults, and evidence exists that using it can interfere with cognitive processes that are central to learning and memory. For example, Martini et al., (2020a, b) showed that vocabulary recall after a 24-h delay was significantly worse when social media was used immediately after learning compared to after a period of quiet wakeful rest (but see Quevedo Pütter & Erdfelder, 2025, who found no effects). Social media use can be conceptualised as an attentionally demanding distraction, as it provides highly personalised and continuously updated content that can capture attention over extended time periods. By replacing a controlled laboratory working memory task with a naturalistic and commonly encountered post-learning activity, our design better approximates the real-world conditions under which memory consolidation may be vulnerable to interference. Thus, Experiment 2 compared the effects of three distinct 7-min post-learning conditions: (1) repetitive listening to the word list (repetition condition), (2) wakeful resting (wakeful resting condition), and (3) engaging in social media use (social media condition).

### Method

#### Participants

Sixty-eight participants took part in the experiment in exchange for course credit. Four participants only completed Session 1. Missing recall data from these four participants for Session 2 were imputed (van Buuren & Groothuis-Oudshoorn, 2011; see Experiment 1). Three participants were excluded from analyses due to failing to achieve a minimum memory performance of 10% on the first recall. The analyzed data set consisted of 65 participants (43 female, 22 male; mean age = 21.9 years, *SD* = 1.8 years, age range = 19–28 years). None of the participants in Experiment 2 reported having taken part in Experiment 1. All participants gave informed consent to take part in the study.

#### Materials and procedure

In the distractor condition of Experiment 2, participants engaged with social media for 7.5 min (see Fig. 1). At the beginning of the experiment, we asked participants whether (a) Facebook, TikTok, and/or Instagram is installed on their mobile phone, (b) they have compatible headphones, and (c) the mobile phone is charged and set to silent mode. To minimize distraction, we asked participants to place their mobile phones behind a partition to their left or right, out of sight to reduce distraction. Participants were instructed to use social media platforms as they would in their everyday lives. They were permitted to switch freely between the platforms and to engage with content by viewing photos and videos, creating posts, and writing comments. However, they were explicitly advised not to follow external links.

At the beginning of the social media condition, immediately following word-learning participants received a message signalling them to start using social media for approximately 7 min. During this period, the computer screen remained black. The end of the social media condition was signalized by a white-black three times flickering screen. Following this signal, participants removed their headphones, placed them along with their mobile phones behind the partition, and proceeded with the working memory task (Fig. 1). The repetition condition and wakeful resting condition were equal to Experiment 1.

### Results

**Fig. 3:**
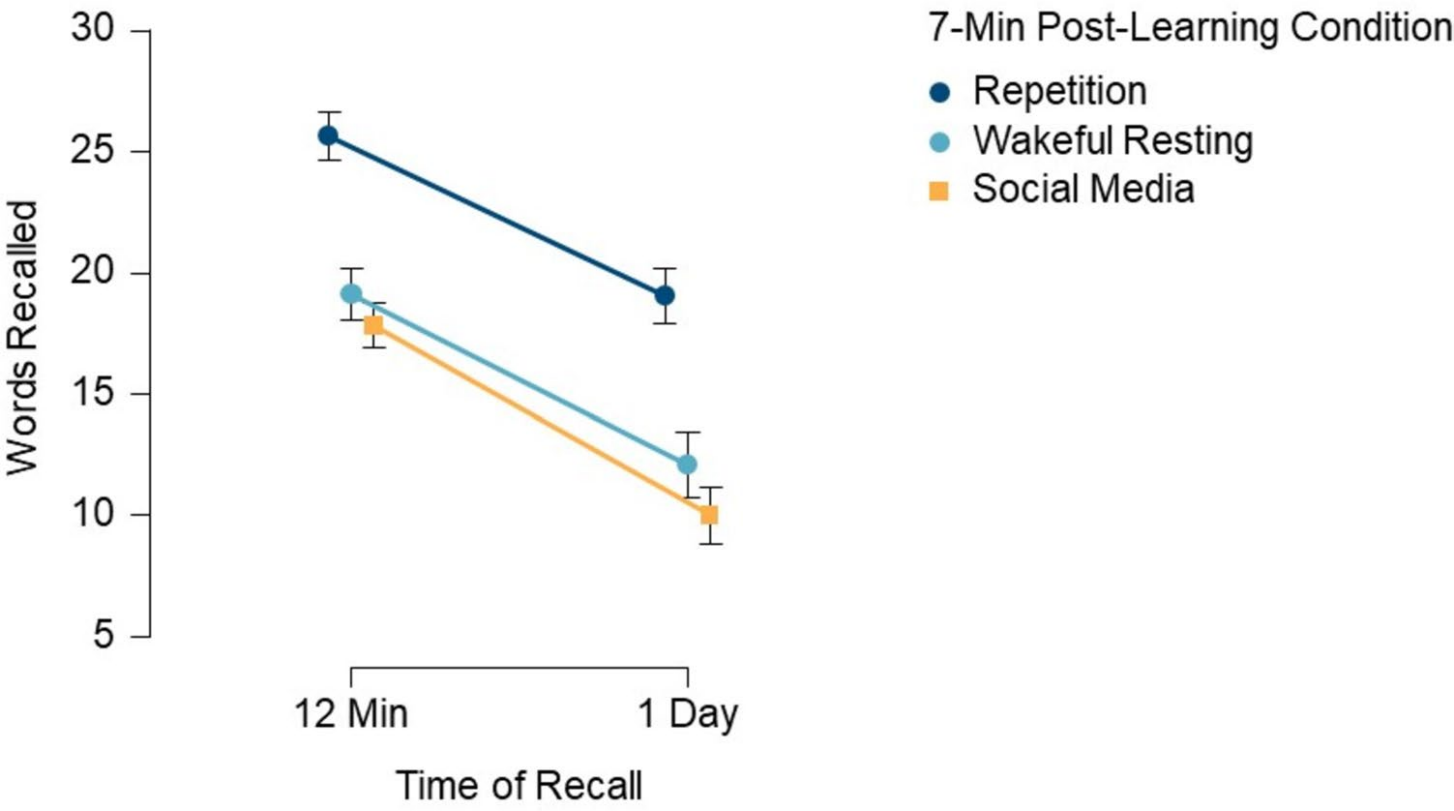
Results from Experiment 2. Mean number of correctly recalled words (maximum = 30 words) as a function of post-learning condition (repetition vs. wakeful resting vs. social media) and time of recall (12 min vs. 1 day). Error bars represent 95% confidence intervals

Figure 3 shows the mean memory performance at the two recall times across the three post-learning conditions, presenting the raw number of words correctly recalled.

A 3 × 2 repeated-measures ANOVA was conducted with the within-subject factors of Post-Learning Condition (repetition, wakeful resting, social media) and Time of Recall (12 min, 1 day). Participants’ memories for the words differed significantly between the three post-learning conditions, *F*(1.8, 116.7) = 89.45, *p* <.001, *η*_p_ =.58 (Greenhouse–Geisser sphericity correction). Contrast analyses revealed that participants retained significantly more words in the repetition condition compared to the wakeful resting condition, *t*(64) = 8.95, *p* <.001, *d* = 1.08, and significantly more words in the wakeful resting condition compared to the social media condition, *t*(64) = 2.55, *p* =.013, *d* = 0.27 (Fig. 3). Retention dropped significantly in all three post-learning conditions over the 1-day delay, *F*(1, 64) = 194.72, *p* <.001, *η*_p_ =.75. Memory performance in the three post-learning conditions did not interact with the time of recall, indicating that memory performance differences between the three post-learning conditions were sustained across the 1-day delay, *F*(2, 128) = 1.33, *p* =.269, *η*_p_ =.020. ANOVA results without imputed data can be found in Table S1 (Online Supplementary Material).

#### Subjective ratings

Participants’ subjective ratings of how often they thought about the words differed significantly between the three post-learning conditions, *Χ*^2^(2) = 72.82, *p* =  <.001, Kendall’s W =.56. Conover’s post hoc comparisons revealed that ratings were higher in the repetition condition (*M* = 4.1, *SD* = 1.2) than in the wakeful resting condition (*M* = 2.8, *SD* = 1.3), *T*(128) = 7.12, *p*_holm_ <.001. Ratings were significantly higher in the repetition condition compared to the social media condition (*M* = 1.6, *SD* =.8), *T*(128) = 12.74, *p*_holm_ <.001, and higher in the wakeful resting condition compared to the social media condition, *T*(128) = 5.62, *p*_holm_ <.001.

Correlations between participants’ subjective ratings and recall performance after 12 min in the respective post-learning condition were small to medium (repetition condition: *r*_Spearman_ =.18, *p* =.15; wakeful resting condition: *r*_Spearman_ =.25, *p* =.049; social media condition: *r*_Spearman_ =.19, *p* =.121). Correlations between participants’ subjective ratings and recall performance after 1 day in the respective post-learning condition were small to medium (repetition condition: *r*_Spearman_ =.31, *p* =.012; wakeful resting condition: *r*_Spearman_ =.32, *p* =.009; social media: *r*_Spearman_ =.28, *p* =.024).^1^

### Discussion

The present study contrasted the effects of three post-learning interventions – repetition, wakeful resting, and distraction with a new task – on the maintenance of a word list over 1 day. Our results showed that additional repetitions of a word list brought a clear memory advantage compared to wakeful resting and distraction. Wakeful resting, in turn, was more beneficial than engaging in a new task. These findings show that the activity that follows learning plays a crucial role in shaping the retention of new memories.

One explanation for our findings is that the opportunity to rehearse the words differed between the three conditions. Accordingly, the opportunity to rehearse words was severely limited in the distractor conditions, resulting in the lowest memory performance. In the wakeful resting condition, rehearsal was possible, but only for those words that could be remembered immediately after learning. This may have strengthened some items that already had high activation in memory, but not those that had low activation in memory. In the repetition condition, the words were presented repetitively – a form of auditory external rehearsal. This repetitive presentation of the word list would allow all words to be strengthened, regardless of their initial strength in memory.

Another explanation for our findings is that forgetting is driven by retroactive interference, that is the accessibility of previous memory traces suffers from the creation of new memory traces (e.g. Wixted, 2004, 2022). Accordingly, it can be argued that during the distractor conditions, i.e. during the working memory task and social media usage, a lot more new memories are encoded than during the other two post-learning conditions, thereby causing a rather large amount of retroactive interference that could cause forgetting of the learned. In the wakeful resting condition, no interfering events are happening, thereby reducing the amount of interference to a minimum. However, memory traces also cannot be strengthened, which is only possible in the repetition condition. Here, the encoded events potentially do not interfere with the already encoded memory traces but can be used to strengthen the existing memory traces and make them more accessible during the following recall tasks.

Our finding that repeated presentation of learning information leads to better memory performance compared to information that is repeated less frequently, as in the wakeful rest and distractor conditions, is consistent with existing findings in the repetition literature (Deese, 1960; Gregg, 1976; Kahana et al., 2024; Popov & Reder, 2020; Ward et al., 2003). Assumptions from the ‘word frequency’ literature suggest that one factor contributing to the high-frequency recall advantage – where frequently presented words are better remembered – is the increased rehearsal of more frequently presented words (Tan & Ward, 2000; Ward et al., 2003). Our findings that participants’ subjective ratings of thinking about the words were highest in the repetition condition and correlated positively with memory performance support this notion. However, these correlations were moderate to low, indicating that rehearsal may not be the only factor of the repetition effect. Furthermore,‘thinking of a word ‘ cannot necessarily be equated with the idea that participants are engaged in explicit rehearsal. Instead, thinking about a memorandum has more often been associated with the concept of attentional refreshing, where refreshing refers to the attentional focussing on memory representations (Bartsch et al., 2018). Alternatively, factors such as the degree of memory consolidation in that repetition can accelerate memory consolidation processes (Kukushkin et al., 2022; Yu et al., 2024) or the use of additional memory strategies (e.g., trying to link words together) might underlie the superior memory performance in the repetition condition.

Our results further showed that wakeful resting promoted memory retention compared to performing a distracting new task. This finding aligns with other wakeful resting studies (Craig et al., 2015; Dewar et al., 2012; Martini et al., 2020a, b; Mercer, 2015). Although explicit rehearsal as an explanation for why resting leads to better memory performance is an obvious explanation for its effect on memory – as explicit rehearsal can be easily induced during rest – existing studies tend to show that the effects of resting on memory might be better explained by the effects of facilitated memory consolidation and reactivation processes during a resting period (Mednick et al., 2011; Tambini & Davachi, 2019; Wamsley, 2019, 2022). This assumption is based on studies using methods designed to minimise rehearsal effects – such as incidental learning tasks (Craig & Dewar, 2018), the use of non-recallable nonwords (Dewar et al., 2014), or neuroscientific analyses of rehearsal activity (Tambini et al., 2010). For example, Dewar et al., (2014, Experiment 2) showed that recognition memory for non-recallable nonwords (e.g., ‚ ‘toijcunn’) was significantly improved following a brief period of wakeful resting compared to performing a visual spot-the-difference task immediately after learning. In our study, although participants’ subjective ratings of how often they thought about the words during wakeful resting showed correlations with recall performance at both testing times, this should not be interpreted as evidence that the memory benefits of wakeful resting were driven by intentional rehearsal. First, recently encountered stimuli can occur spontaneously in consciousness during post-encoding rest periods, with an increasing likelihood of stimulus intensity (Müller & Pilzecker, 1900). Our word lists were presented three times each, which may have led to a stronger memory representation and increased the likelihood of capturing participants’ attention (attentional refreshing). Second, the correlation coefficients were moderately low, suggesting that mentation about the words was not the driving factor for the wakeful resting effect (see Tambini & Davachi, 2019; Wamsley, 2019). Third, in our study we did not include an immediate post-learning memory test. It cannot be ruled out that participants had a higher expectation of an upcoming memory test, which led them to think about the words more often. The reason why we excluded an immediate memory test after learning was to minimise the memory-enhancing effects of active retrieval, as active retrieval has been shown to accelerate memory consolidation processes (e.g., Antony et al., 2017; Rowland, 2014). It is likely that intentional and spontaneous rehearsal of previously learned information during a wakeful resting period promotes memory retention in addition to facilitating memory consolidation processes.

Another finding was that the memory-modulating effects of the post-learning interventions persisted for one day. Memory performance declined significantly across all three conditions; however, the relative memory performance between the three conditions remained consistent (Dewar et al., 2012). This uniform decline in memory performance indicates that only the most stable memory traces were preserved over time, while weaker representations were lost. This pattern of results is consistent with the synaptic downscaling hypothesis, whereby the strength of synapses is reduced during sleep while relative differences in synaptic strength, and therefore memory traces, are preserved (Cirelli & Tononi, 2022; Tononi & Cirelli, 2003). Synaptic downscaling would account for the general decline in memory strength while maintaining the relative strength induced by the different post-learning conditions.

Further research is required contrasting the effects of wakeful resting and repetition on memory retention. Questions that remain include how many repetitions provide an advantage over wakeful resting and whether wakeful resting might offer a memory benefit over repetition in certain cases – for example, when the learning material becomes more complex or extensive.

In conclusion, our study is the first to directly compare the effects of wakeful resting and repetition on memory retention. While wakeful resting is a relatively simple and easy to implement strategy to improve memory, our results suggest that repetition offers a clear advantage in terms of memory performance when tested directly.

## Supplementary Information

Below is the link to the electronic supplementary material.

Supplementary file1 (DOCX 17 kb)

## Data Availability

Data and materials are available via the Open Science Framework at: https://osf.io/pa9qy/

## Footnotes

1 Combined analyses of Experiments 1 and 2 showed that participants reported thinking most about the words in the repetition condition, followed by the wakeful resting condition, and least in the distractor conditions. Correlational analyses of the combined data from Experiments 1 and 2 also showed that in all three post-learning conditions, higher self-reported ratings of thinking about the words were significantly correlated with recall performance after 12 min and after 1 day.
