# Supplementary material for: Resting after learning or repeating the learned?
Source: Psychon Bull Rev. 2025 Dec 15;33(1):15. doi: 10.3758/s13423-025-02820-4 (PMC12705850; doi:10.3758/s13423-025-02820-4)
Supplement: Supplementary file 1 — Supplementary file1 (DOCX 17 kb) [file 13423_2025_2820_MOESM1_ESM.docx]

Supplement

Table S1

ANOVA analysis excluding imputed data.

|  | Experiment 1 |  | Experiment 2 |
| --- | --- | --- | --- |
| *Within Subjects Effects* |  |  |  |
| Time of Recall (12 min vs. 1 day) | *F*(1,35) = 145.97, *p* < .001, *η*^2^_p_ = .81 |  | *F*(1,60) = 235.54, *p* <. 001, *η*^2^_p_ = .80 |
| Post-Learning condition (repetition vs. wakeful resting vs. distraction^1^) | *F*(2,70) = 20.53, *p* < .001, *η*^2^_p_ = .37 |  | *F*(2,120) = 79.97, *p* < .001, *η*^2^_p_ = .57 |
| Time of Recall * Post-Learning Condition | *F*(2,70) = 0.98, *p* = .380, *η*^2^_p_ = .03 |  | *F*(2,120) = 1.36, *p* = .261, *η*^2^_p_ = .02 |
| *Repeated contrasts* |  |  |  |
| repetition - wakeful resting | *t*(35) = 4.59, *p* < .001, *d* = 0.62 |  | *t*(60) = 8.57, *p* < .001, *d* = 1.09 |
| wakeful resting - distraction | *t*(35) = 1.99, *p* = .055, *d* = 0.31 |  | *t*(60) = 2.23, *p* = .030, *d* = 0.25 |

^1^ working memory task (Experiment 1), social media usage (Experiment 2).

*Note.* Table S1 shows the results of the repeated measures ANOVA analysis of participants' memory performance in Experiments 1 and 2 excluding imputed data (see Method section). The changes in the results are potentially due to a decrease in power and an increased imbalance of participants in the post-learning order conditions. Combined contrast analyses of Experiments 1 + 2 excluding imputed data revealed that participants retained significantly more words in the repetition condition than in the wakeful resting condition, *t*(96) = 9.50, *p* < .001, *d* = 0.91, and significantly more words in the wakeful resting condition than in the distractor conditions, *t*(64) = 3.0, *p* = .003, *d* = 0.27.
